# Targeted inhibition of KDM6 histone demethylases eradicates tumor-initiating cells via enhancer reprogramming in colorectal cancer

**DOI:** 10.7150/thno.47081

**Published:** 2020-08-08

**Authors:** Junbao Zhang, Ying Ying, Meiqi Li, Maolin Wang, Xiaoyan Huang, Min Jia, Junhui Zeng, Canjie Ma, Yixiang Zhang, Chen Li, Xiaomei Wang, Xing-sheng Shu

**Affiliations:** 1 Department of Physiology, School of Medicine, Health Science Center, Shenzhen University, Shenzhen 518060, China.; 2 Department of Urology, The Second Affiliated Hospital of Jinan University, Shenzhen People's Hospital, Shenzhen 518020, China.; 3 Department of Oncology, Peking University Shenzhen Hospital, Shenzhen Key Laboratory of Gastrointestinal Cancer Translational Research, Cancer Institute of Shenzhen-PKU-HKUST Medical Center, Shenzhen 518036, China.

**Keywords:** GSK-J4, KDM6, tumor-initiating cells, super-enhancer, colorectal cancer

## Abstract

Tumor-initiating cells (TICs) maintain heterogeneity within tumors and seed metastases at distant sites, contributing to therapeutic resistance and disease recurrence. In colorectal cancer (CRC), strategy that effectively eradicates TICs and is of potential value for clinical use still remains in need.

**Methods**: The anti-tumorigenic activity of a small-molecule inhibitor of KDM6 histone demethylases named GSK-J4 in CRC was evaluated by *in vitro* assays and *in vivo* imaging of xenografted tumors. Sphere formation, flow cytometry analysis of cell surface markers and intestinal organoid formation were performed to examine the impact of GSK-J4 on TIC properties. Transcriptome analysis and global profiling of H3K27ac, H3K27me3, and KDM6A levels by ChIP-seq were conducted to elucidate how KDM6 inhibition reshapes epigenetic landscape and thereby eliminating TICs.

**Results**: GSK-J4 alleviated the malignant phenotypes of CRC cells *in vitro* and *in vivo*, sensitized them to chemotherapeutic treatment, and strongly repressed TIC properties and stemness-associated gene signatures in these cells. Mechanistically, KDM6 inhibition induced global enhancer reprogramming with a preferential impact on super-enhancer-associated genes, including some key genes that control stemness in CRC such as *ID1*. Besides, expression of both Kdm6a and Kdm6b was more abundant in mouse intestinal crypt when compared with upper villus and inhibition of their activities blocked intestinal organoid formation. Finally, we unveiled the power of KDM6B in predicting both the overall survival outcome and recurrence of CRC patients.

**Conclusions**: Our study provides a novel rational strategy to eradicate TICs through reshaping epigenetic landscape in CRC, which might also be beneficial for optimizing current therapeutics.

## Introduction

Tumor heterogeneity originated from tumor-initiating cells (TICs) has been widely considered as one of the main causes of therapeutic resistance and disease recurrence 1, 2. Alterations of intestinal stem cells (ISCs) residing at the crypts of small intestine and colon epithelium drives the initiation and propagation of neoplastic lesions, which eventually give rise to intestinal cancers including colorectal cancer (CRC) 3. Thus, TICs of CRC shares certain common genetic and epigenetic features with ISCs, and are sometimes termed as colorectal cancer stem cells (CSCs) 4, 5. The essential role of TICs in CRC initiation and progression was best illustrated by recent studies using elegant lineage-tracing strategies, which demonstrated that targeted elimination of TICs sustains primary CRC growth and abolishes the capacity of CRC cells to establish liver metastases 6, 7. However, most of the current therapeutic regimens in clinical use for CRC do not show ideal effect against TICs that usually stay quiescent and reserve strong potential of plasticity, urging the demand for novel strategy targeting TICs.

Post-translational modifications of histones play critical roles in shaping chromatin architecture and regulating gene transcription. During embryogenesis, fine tuning histone H3 tri-methylation at lysine 27 (H3K27me3) level is crucial to proper temporospatial expression of genes determining cell fate transition 8. The cellular dynamics of H3K27me3 is achieved through precise control of the activities of both polycomb complex 2 (PRC2) that writes this code and lysine demethylase 6 (KDM6) family proteins that specifically removes it 9, 10. Importantly, distorted H3K27me3 landscape due to alterations in PRC2 subunits and two KDM6 enzymes (KDM6A or UTX, KDM6B or JMJD3) has been observed in the vast majority of human cancers 11-13. Thus, therapeutic potential of counteracting hyper PRC2 activity, such as EZH2 inhibitors, in cancer has been under intensive investigation for the past decades 14, whereas strategies targeting KDM6 in cancer treatment that just emerged in recent few years surprisingly have already demonstrated promising effect against various cancer types, including glioma 15-17, neuroblastoma 18, acute myeloid leukemia 19, and castration resistant prostate cancer 20. Nonetheless, the mechanisms underlying KDM6 inhibition-mediated tumor suppression are still poorly understood. On the other hand, although it is clear that KDM6A and KDM6B are important for fate determination of embryonic stem cells (ESCs) during development 9, whether they are required for sustaining TICs activity in tumorigenesis remains elusive.

To fill these vacancies, this study evaluated the therapeutic potential of a small-molecule KDM6 inhibitor, GSK-J4, in CRC, explored its power in eradicating TICs, and proposed a model delineating how KDM6 inhibition reshapes chromatin environment via enhancer reprogramming at key genes controlling stemness. In addition, we also unveiled the prognostic value of *KDM6B* expression in predicting CRC recurrence.

## Results

### GSK-J4 weakens malignant phenotypes of CRC cells and sensitizes them to chemotherapeutic treatment

**Figure 1:**
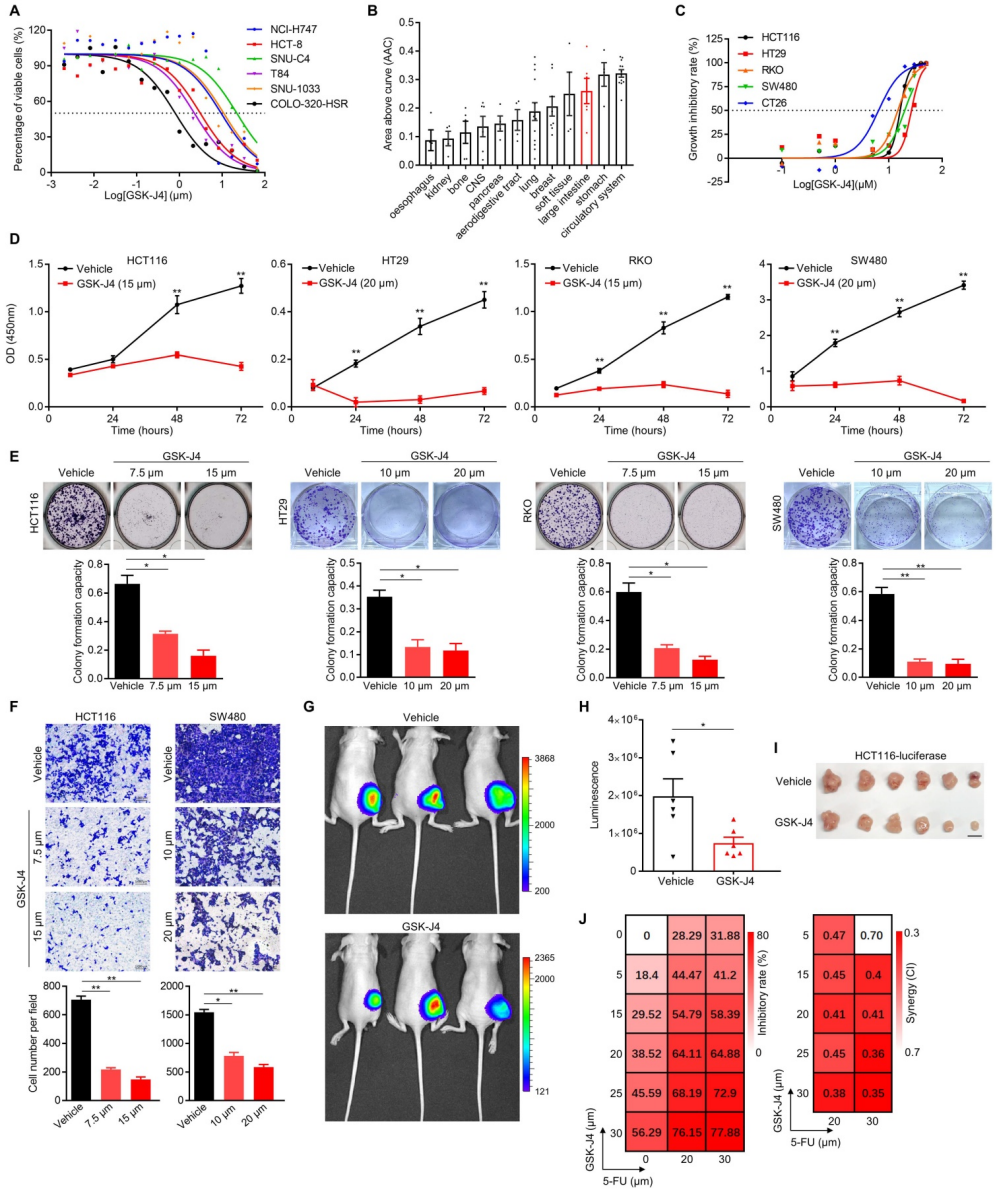
** GSK-J4 weakens malignant phenotypes of CRC cells and sensitizes them to chemotherapeutic treatment.** (**A**) Dose-response curves of GSK-J4 in six CRC cell lines. Dashed line represents an inhibitory rate of 50%. (**B**) Area above cure (AAC) of GSK-J4 dose-response curves in cell line from different tissue origins. Source data of (A) and (B) were from PharmacoDB database. (**C**) Dose-response curves of GSK-J4 in four human CRC cell lines and one murine CRC cell line CT26 determined by cell proliferation assay. Dashed line indicates an inhibitory rate of 50%. (**D**) Cell proliferation assay for CRC cells treated with specified dose of vehicle DMSO or GSK-J4 at indicated time points. (**E**) Colony formation assay for CRC cells treated with specified dose of vehicle DMSO or GSK-J4. For each cell line, one higher dose that was close to its IC50 concentration of GSK-J4 and one lower dose taken as half of the former dose were used. (**F**) Transwell migration assay in CRC cells treated with or without GSK-J4 at different doses. (**G**) *In vivo* imaging of HCT116-luc tumor-bearing mice treated with or without 25 mg/kg GSK-J4 every two days. Luminescence was imaged on day 10 after first treatment. Color key indicating the range of luminescence signal in each photo was shown at right. (**H**) Quantification of the luminescence signals described in (G). (**I**) Photo of tumors dissected from mice described in (G). Scale bar represents 1 cm. (**J**) Heatmaps showing inhibitory rate and combinatory index (CI) across five doses of GSK-J4 and two doses of 5-FU in HCT116 cells. Mean values of triple replicates were shown. CI were calculated by CompuSyn software (http://www.combosyn.com/). In general, a CI smaller than 0.6 was considered as high synergy and a CI smaller than 0.4 was considered as strong synergy. * *p* < 0.05; ** *p* < 0.01; n.s., non-significant.

In an effort to search for potential small-molecule drugs targeting epigenetic modifiers including histone demethylases for CRC treatment, we identified GSK-J4, a potent cell-permeable inhibitor blocking the catalytic site of both KDM6A and KDM6B, effectively suppressed the proliferation of CRC cells from various origins (IC50 ranging from 0.75 µm to 21.41 µm with a median of 6.20 to 6.35 µm) according to data from PharmacoDB database 21 (Figure 1A). Moreover, cell lines derived from large intestine responded better to GSK-J4 than the ones of most other tissue origins (Figure 1B). Similar results were observed in another four human CRC cell lines and one mouse CRC cell line CT26 in our detection system (Figure 1C). We further showed that GSK-J4 inhibited CRC cell proliferation in a time-dependent manner (Figure 1D). Consistently, GSK-J4 treatment strongly inhibits both colony formation (Figure 1E and Figure S1A) and migration (Figure 1F and Figure S1B) capacity of CRC cells and significantly constrained subcutaneous CRC tumor growth *in vivo* (Figure 1G-I). Furthermore, we found that CRC cells were sensitized to a common chemotherapeutic regimen 5-FU (fluorouracil) by GSK-J4 treatment through showing that combinatory administration of these two chemicals synergistically inhibited cell proliferation (Figure 1J). These findings indicate that GSK-J4 holds potential for application in CRC treatment, thus we continued to investigate its mechanism-of-action in tumor suppression.

### GSK-J4 strongly represses TIC properties in CRC

**Figure 2:**
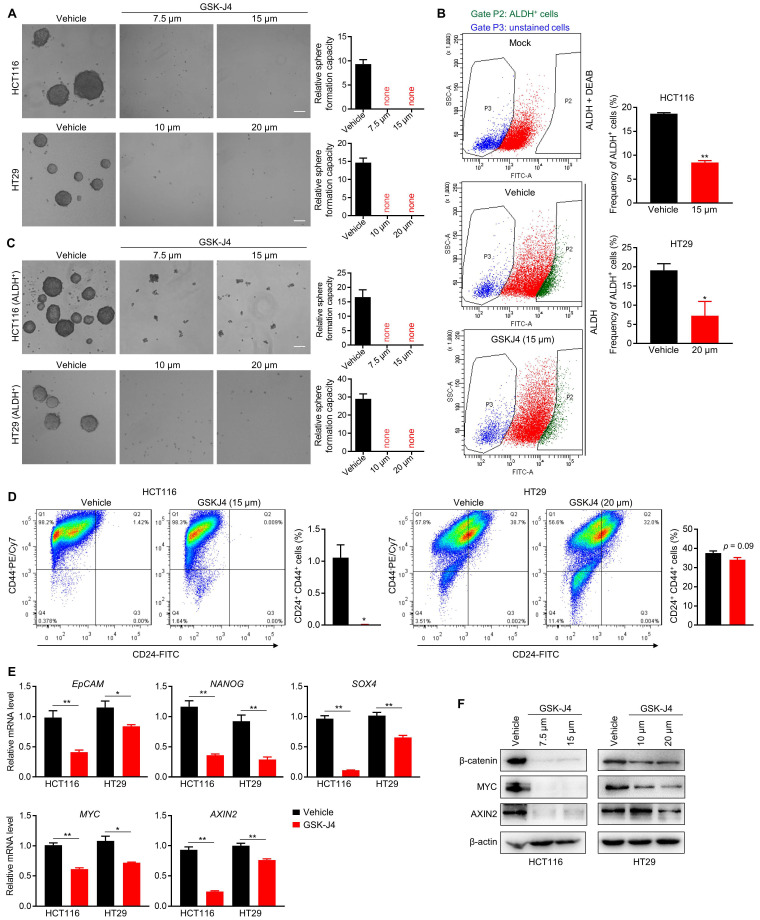
** GSK-J4 strongly represses TIC properties in CRC.** (**A**) Sphere formation assay in HCT116 and HT29 cells treated with or without GSK-J4 at indicated doses. Scale bar represents 100 µm. (**B**) Left panel: representative results of ALDH activity assay in HCT116 cells treated with or without GSK-J4 as determined by flow cytometry. A group of mock cells incubated with both activated ALDH reagent and its inhibitor DEAB was introduced as background control. Cells in Gate P2 were defined as ALDH positive cells. Right panel: quantification of the percentages of ALDH positive cells from flow cytometry analysis in HCT116 and HT29 cells. (**C**) Sphere formation assay in ALDH-positive HCT116 and HT29 cells treated with or without GSK-J4 at indicated doses. Scale bar represents 100 µm. (**D**) Flow cytometry analysis of CD24^+^CD44^+^ cells (upper-right quadrant) with or without GSK-J4 treatment for 48 h. (**E**) qRT-PCR analysis of the expression of indicated genes in HCT116 cells treated with or without 15 µm GSK-J4 and in HT29 cells treated with or without 20 µm GSK-J4 for 48 h. (**F**) Western blotting results showing the expression of total β-catenin, AXIN2 and MYC in HCT116 and HT29 cells treated with or without GSK-J4 at indicated doses. β-actin was used as a loading control. * *p* < 0.05; ** *p* < 0.01.

We next sought to study whether inhibition of KDM6A and KDM6B activities could impair the TIC properties of CRC cells as they were found to be indispensable for the maintenance of TICs' identity and function in some other tumors 22-24. Indeed, even at a low dose close to IC20, GSK-J4 severely inhibited the sphere formation ability of CRC cells (Figure 2A) and reduced the proportion of ALDH^+^ cells that are normally considered as TIC-like cells in the total cell population (Figure 2B). Moreover, sphere formation capacity of ALDH^+^ CRC cells were also greatly suppressed by GSK-J4 (Figure 2C). Similarly, GSK-J4 treatment also decreased the proportion of CD24^+^CD44^+^ stem-like cells (Figure 2D). In line with these results, GSK-J4 downregulated several key signature genes of TICs (Figure 2E) and strongly decreased total β-catenin and MYC protein level (Figure 2F) in CRC cells. Taken together, these findings suggest that KDM6A and KDM6B inhibition impairs the stemness of TICs in CRC.

### Kdm6a and Kdm6b are expressed more abundantly in mouse intestinal crypt than in villus and are required for intestinal organoid formation

**Figure 3:**
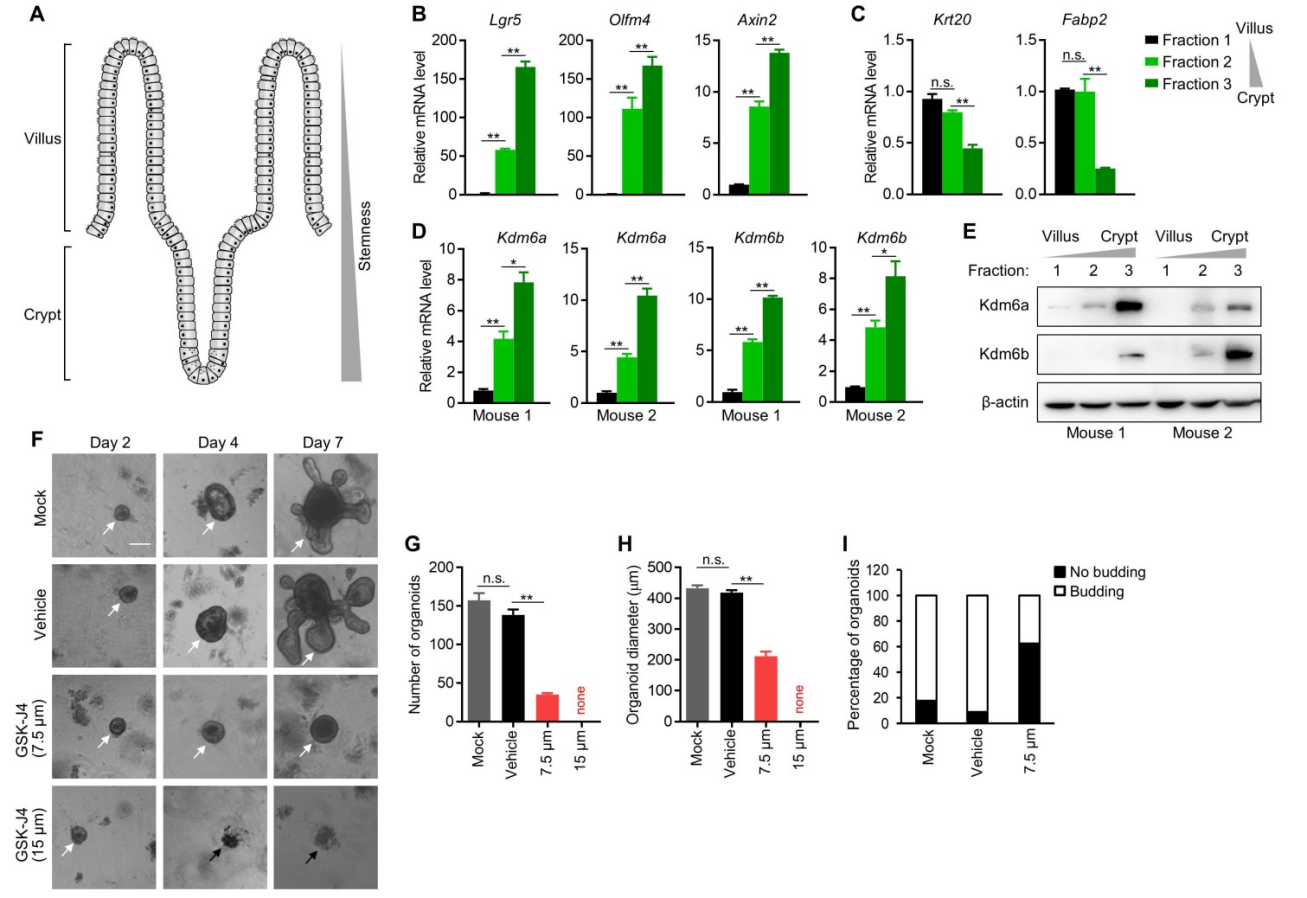
** Kdm6a and Kdm6b are expressed more abundantly in mouse intestinal crypt than in villus and are required for intestinal organoid formation.** (**A**) Diagram showing the structure of typical intestinal villi. ISCs with strong stemness are located at bottom crypts and upper villus contains differentiated enterocytes and secretory cells. (**B-D**) Expression level of indicated genes was determined by qRT-PCR in three fractions of epithelium samples isolated from mouse small intestine. (**E**) Western blotting analysis of Kdm6a and Kdm6b levels in three fractions of epithelium samples isolated from mouse small intestine. β-actin was used as a loading control. (**F**) Representative results of intestinal organoid formation assay with or without GSK-J4 treatment at indicated doses. Images were taken at day 10 after seeding crypts. White arrows indicate viable organoids, and black arrows represents dead organoids. Scale bar represents 100 µm and applies to all images here. Quantifications of organoid number (**G**), diameter (**H**), and morphology type (round cystic only or budding) (**I**) were conducted for organoid formation experiments described in (F). * *p* < 0.05; ** *p* < 0.01; n.s., non-significant.

The intestinal villi maintain high turnover rate of the epithelium by ISCs residing in the crypt of each villus 25, which demonstrate remarkable multipotency and thus fully support the regeneration of differentiated enterocytes and other secretory cells at upper villus (Figure 3A). Importantly, ISCs share certain common features with TICs in intestinal cancer, and are considered as potential cells-of-origin during early dysplasia. Therefore, we speculated whether KDM6A and KDM6B also play a role in ISCs which might explain, at least in part, the dependency of CRC TICs on them. To this end, we isolated mouse intestinal epithelium into three fractions (fraction 1 to 3) with differentiated enterocytes most enriched in fraction 1 and crypts most enriched in fraction 3 according to visual examination under a light microscope. We further confirmed the identities of these three fractions by showing their correlation with the expression of several key stemness marker genes (*Lgr5*, *Olfm4* and *Axin2*) (Figure 3B) and differentiated enterocytes marker genes (*Krt20* and *Fabp2*) (Figure 3C). Using this system, we found the expression of both Kdm6a and Kdm6b were significantly higher in intestinal crypts compared to upper villus (Figure 3D-E), implying their possible role in maintaining ISCs. Thus, we next evaluated the effect of inhibiting Kdm6a and Kdm6b in intestinal organoid formation which is driven by ISCs in the crypts. The results showed that GSK-J4 treatment dramatically reduced the organoid formation efficiency (Figure 3F-G) and repressed organoid growth (Figure 3H) and budding of crypt-like structure from the organoid body (Figure 3I). These evidences suggest a potential role of Kdm6a and Kdm6b in ISCs function which might contribute to TICs formation and/or maintenance during tumorigenesis.

### GSK-J4 downregulates stemness-associated gene signatures in CRC

**Figure 4:**
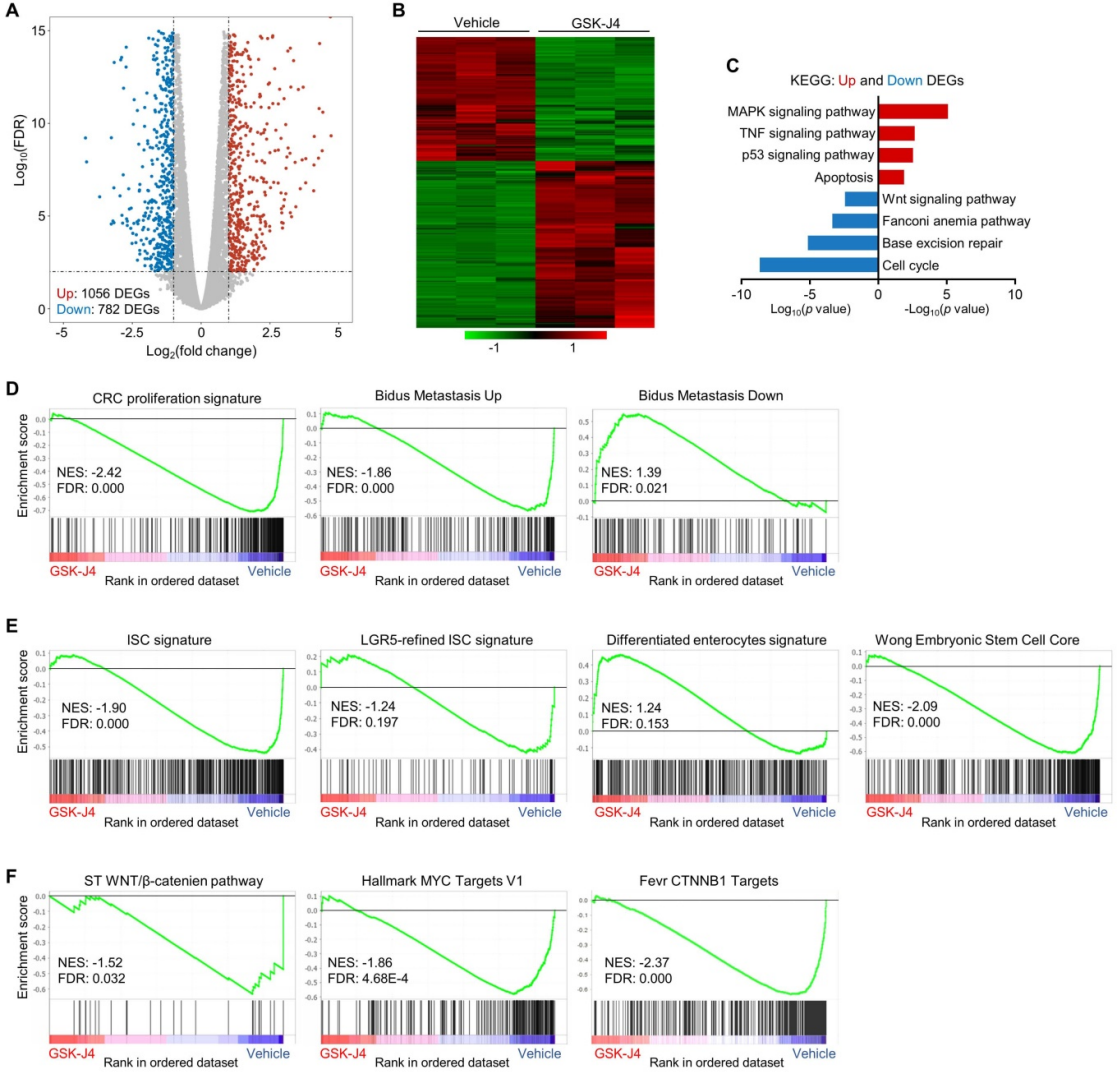
** GSK-J4 downregulates stemness-associated gene signatures in CRC.** (**A**) Volcano plot showing differentially expressed genes (DEGs) from RNA-seq analysis of HCT116 cells treated with or without 15 µm GSK-J4 for 48 h. Genes with fold change ≥ 2 and false discovery rate (FDR) ≤ 0.01 were considered as significant DEGs. (**B**) Heatmap showing expression level of DEGs described in (A) across indicated samples. (**C**) Results of Kyoto Encyclopedia of Genes and Genomes (KEGG) pathway analysis of DEGs described in (A). (**D-F**) Results of GSEA using specified gene sets and transcriptomic data described in (A). FDR ≤ 0.25 were considered as statistically significant. NES, normalized enrichment score.

To explore the molecular mechanism underlying GSK-J4-induced tumor suppression, we analysed global transcriptomic change upon GSK-J4 treatment in CRC cells and identified 782 downregulated differentially expressed genes (DEGs) and 1056 upregulated DEGs (Log_2_ fold-change ≥ 1 and FDR ≤ 0.01) (Figure 4A-B). Pathway enrichment analysis of DEGs showed that downregulated genes were mostly enriched in cell cycle, DNA repair and Wnt signaling pathways, while upregulated DEGs were overrepresented in MAPK, TNF, p53 and apoptosis pathways (Figure 4C). Further gene set enrichment analysis (GSEA) demonstrated that signatures promoting proliferation and metastasis were downregulated by GSK-J4, and genes potentially impede metastasis were upregulated (Figure 4D). Remarkably, KDM6A and KMD6B inhibition greatly decreased the expression of bulk and LGR5-refined ISC signatures (Figure 4E), which have been shown to be able to identify CSCs in CRC and predict CRC recurrence. Concordantly, GSK-J4 also upregulated gene sets associated with differentiated enterocytes and repressed genes essential for ESCs (Figure 4E). Moreover, transcription of Wnt pathway genes as well as its downstream target genes was suppressed upon GSK-J4 treatment (Figure 4F), further confirming reduced cell stemness due to KDM6A and KDM6B inhibition.

### KDM6 inhibition induces enhancer reprogramming at key stemness-controlling genes

**Figure 5:**
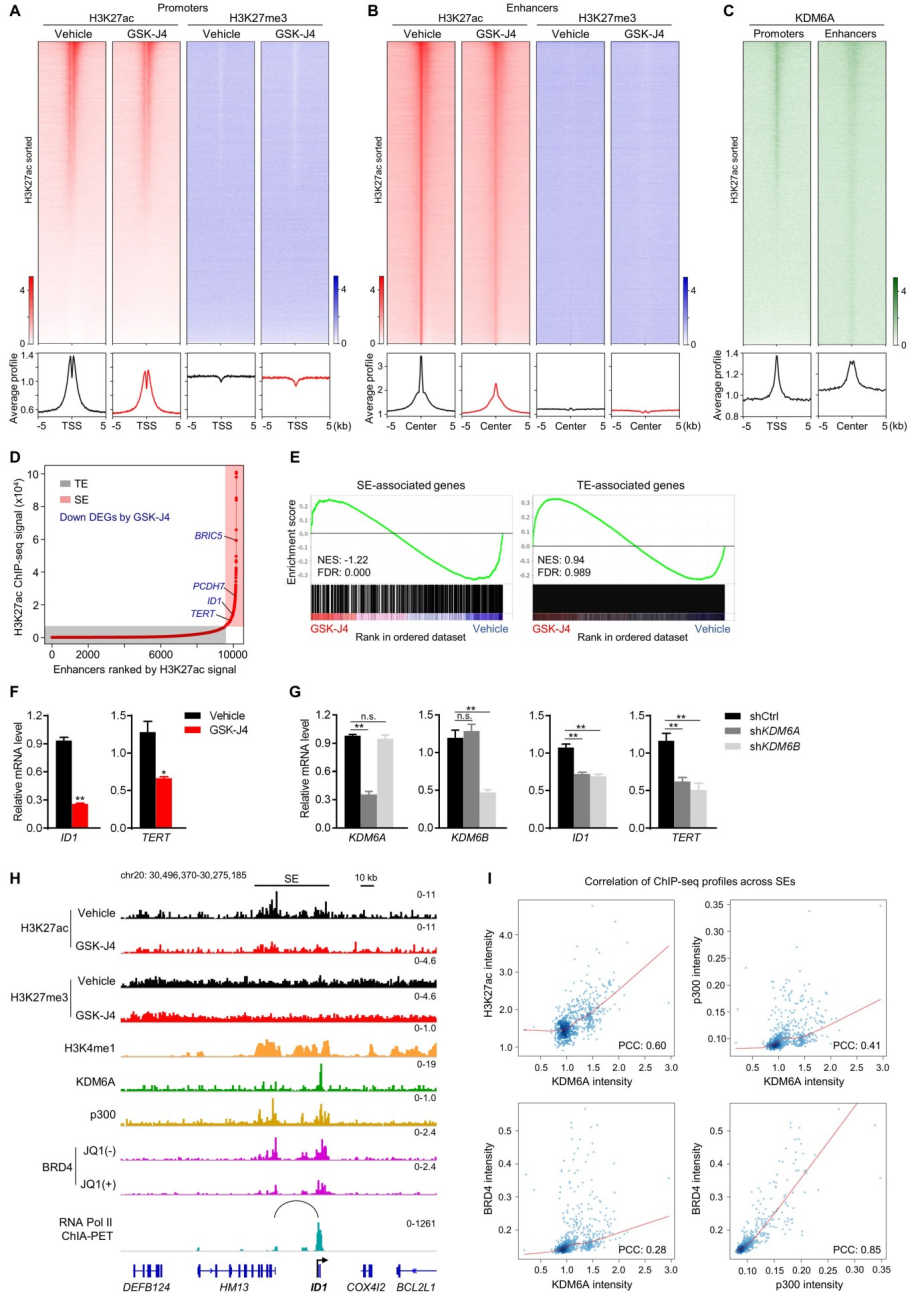
** KDM6 inhibition induces enhancer reprogramming at key stemness-controlling genes.** (**A**) Heatmaps and average profile curves of H3K27ac and H3K27me3 ChIP-seq data across a 10 kb window centered at transcription start sites (TSS) in HCT116 cells. (**B**) Heatmaps and average profile curves of H3K27ac and H3K27me3 ChIP-seq data across a 10 kb window centered at H3K27ac peaks that do not fall into the -2 kb to 1 kb region of any TSS in the genome. (**C**) Left panel: heatmap and average profile curve of KDM6A across the intervals described in (A); Left panel: heatmap and average profile curve of KDM6A across the intervals described in (B). Genomic intervals in all heatmaps were sorted according to the level of H3K27ac signal level in vehicle group. (**D**) Super-enhancer analysis with ROSE algorithm using H3K27ac ChIP-seq data of HCT116 cells treated with vehicle. Several representative DEGs downregulated by GSK-J4 and associated with super-enhancer were denoted. TE, typical-enhancer; SE, super-enhancer. (**E**) GSEA using gene sets annotated to TE or SE in RNA-seq data of HCT116 cells treated with or without 15 µm GSK-J4 for 48 h. NES, normalized enrichment score; FDR, false discovery rate. (**F**) mRNA level of *ID1* and *TERT* were evaluated by qRT-PCR in HCT116 cells treated with or without 15 µm GSK-J4 for 48 h. (**G**) Expression level of indicated genes was examined by qRT-PCR in HCT116 cells 72 h after infection with lentivirus containing shRNA targeting *KDM6A* or *KDM6B*. A non-targeting sequence cloned into the same backbone (shCtrl) was used as control. (**H**) ChIP-seq and ChIA-PET profiles of indicated antibodies at *ID1* gene locus in HCT116 cells as demonstrated by Integrative Genomics Viewer (IGV). The black line represents the span of the super-enhancer (SE) associated with *ID1*. The arc denotes a chromatin interaction identified by RNA Pol II ChIA-PET experiment. (**I**) Scatter plots showing correlation of ChIP-seq signal intensities across all super-enhancers in HCT116 cells. ChIP-seq data of H3K4me1, BRD4 and p300 were from GSE101646, GSE57628 and GSE51176 respectively. ChIA-PET data of RNA Pol II was from GSE39495. PCC, Pearson correlation coefficient. * *p* < 0.05; ** *p* < 0.01; n.s., non-significant.

As KDM proteins participate in shaping chromatin architecture by keeping various histone modifications in a precise balance, we postulated that KDM6A and KDM6B inhibition altered epigenetic landscape at distal or proximal regulatory elements of their target genes, which eventually lead to dysregulation of target gene transcription. To test this hypothesis, we first documented the global profile of histone H3 acetylation at lysine 27 (H3K27ac), a histone mark labelling the activity of both proximal promoters and distal enhancers, in CRC cells with or without GSK-J4 treatment. Surprisingly, the data showed that GSK-J4 decreased the H3K27ac level at both global promoters (Figure 5A, left panel) and enhancers (Figure 5B, left panel). In comparison, we also examined H3K27me3 levels at these sites and did not observe substantial change after GSK-J4 treatment (Figure 5A-B, right panel), although we did detect increased H3K27me3 levels across typical H3K27me3 domains (Figure S2A, left panel) that frequently located at distal intergenic regions (Figure S2B) and showed little overlap with H3K27ac-positive sites (Figure S2A, right panel). Interestingly, KDM6A showed enrichment of binding at H3K27ac-positive promoters and enhancers (Figure 5C), while an antibody of KDM6B we used failed to produce any ChIP-seq signal.

The above results indicated that inhibition of KDM6A and KDM6B mounted a prominent impact on active enhancers, which might further affect the activity of their cognate promoters. Therefore, we next pursued the possible functional consequences of this enhancer reprogramming. Through integrative analysis of our transcriptomic and epigenomic data in the same cell system, we found that most of the super-enhancer (SE) associated genes were significantly downregulated by GSK-J4, such as *BRIC5*, *PCDH7*, *ID1*, and *TERT* (Figure 5D-E). Moreover, compared with typical enhancer (TE) associated genes, SE-associated genes responded more sensitively to GSK-J4 treatment (Figure 5E). We noticed that some of the SE-associated genes repressed by GSK-J4 treatment are well-known critical regulators of cancer cell stemness, such as *ID1* 26, 27 and *TERT* 28, and further confirmed their downregulation by GSK-J4 or shRNAs targeting *KDM6A* or *KDM6B* in two CRC cell lines (Figure 5F-G and Figure S2C-D). Indeed, when looked closely into the *ID1* locus, we found the H3K27ac level was decreased across the SE but the H3K27me3 level largely remained unchanged. Consistently, we also observed enrichment of several other SE signature modification or proteins, including H3K4me1, p300, and BRD4, as well as KDM6A at *ID1* locus. Moreover, JQ1, a BRD4 inhibitor, dramatically reduced BRD4 level within *ID1* locus. Intriguingly, by using chromatin interaction analysis with paired-end tag sequencing (ChIA-PET) data of RNA Polymerase II in the same cell line, we identified a distal active enhancer constituent in the SE that physically contact with *ID1* promoter via higher-order chromatin interaction (Figure 5H). These findings consistently support the conclusion that *ID1* transcription is driven by a SE regulated by KDM6A and potentially by KDM6B in CRC cells. In addition, we found that KDM6A binding intensity correlated well with H3K27ac, p300, and BRD4 intensity levels across all SEs in the genome (Figure 5I), suggesting that KDM6A might be involved in global SE regulation.

### *KDM6B* expression level predicts survival outcome and recurrence of CRC patients

**Figure 6:**
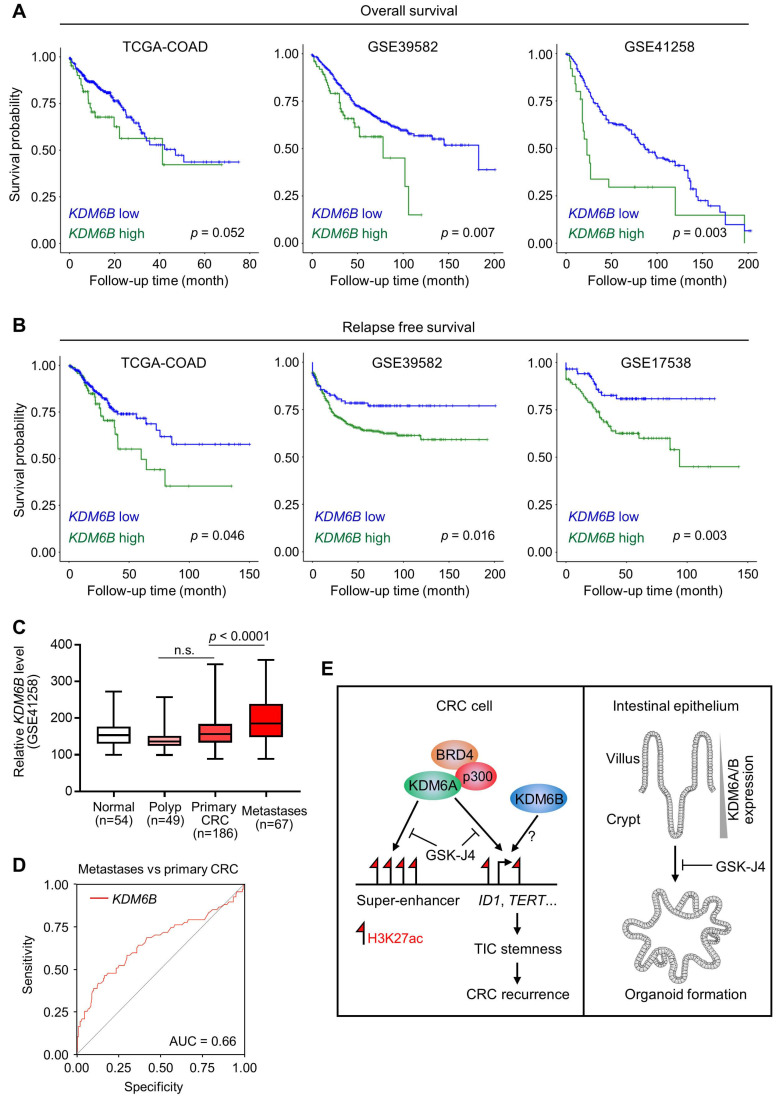
***KDM6B* expression level predicts survival outcome and recurrence of CRC patients.** Kaplan-Meier survival curves showing lower *KDM6B* expression level is associated with both better overall survival (**A**) and better relapse-free survival (**B**) of CRC patients in several independent cohorts. Significance was evaluated by Log-Rank test. (**C**) Expression of *KDM6B* in GSE41258 dataset was shown by boxplots with data range. n.s., non-significant. (**D**) ROC (receiver operating characteristic) curve in GSE41258 dataset. AUC, area under curve. (**E**) Schematic diagram showing major findings in this study. Refer to text for description in detail.

Based on the above findings revealing the importance of KDM6A and KDM6B in CRC development and progression, we then tried to elucidate the relevance of *KDM6* genes expression to clinical outcomes of CRC patients. As a result, we found that higher *KDM6B* expression was significantly associated with poorer overall survival (Figure 6A) and predicted worse recurrence (Figure 6B) of CRC patients from several independent cohorts. In contrast, however, *KDM6A* did not demonstrate consistent prognostic value across different datasets, although its higher expression was associated with better overall survival but more frequent disease relapse in TCGA-CRC cohort (Figure S3A). Interestingly, higher *KDM6B* expression also acted as a predictor for recurrence in some other common cancers, including hepatocellular carcinoma, lung adenocarcinoma, pancreatic cancer, and bladder cancer (Figure S3B). Given that TICs are considered as the cells that seed distant metastases and a substantial part of CRC recurrence is associated with metastasis, we next examined whether *KDM6B* expression was elevated in metastases when compared with primary tumors and observed a result as expected in a CRC dataset containing both tissue specimens (Figure 6C). Moreover, ROC (receiver operating characteristic) curve in the same dataset showed that *KDM6B* expression level by itself can identify a major part of metastases from primary CRC tumors (AUC = 0.66) (Figure 6D). Based on clinical settings, these results further consolidate the therapeutic rationale targeting KDM6 enzymes in CRC and also pinpoint *KDM6B* as a potential predictor for recurrence of CRC and other common cancers.

## Discussion

In this study, we reported that targeted inhibition of histone demethylases KDM6A and KDM6B simultaneously by GSK-J4 suppressed CRC growth and progression through eliminating TICs. Mechanistically, we unveiled a novel function of GSK-J4 in reprogramming super-enhancers at key stemness-controlling genes such as *ID1*, which might be at least partially maintained by cooperative action of KDM6A with transcription coactivators p300 and BRD4 (Figure 6E, left panel). Besides, although we failed to explain the role of KDM6B in shaping enhancers in our system due to technical problems as mentioned above, we provided evidence about its regulatory potential at the same target genes controlled by KDM6A, thus whether KDM6A and KDM6B play redundant or differential role at these sites worth further study in the future. On the other hand, we found that both KDM6A and KDM6B were enriched in intestinal crypts when compared with upper villus which correlated with increased stemness of epithelium cells from top to bottom along the villus, and intestinal organoid formation was strongly inhibited by GSK-J4, implying possible roles of these two enzymes in regulating adult ISCs (Figure 6E, right panel).

It is widely accepted that CRC originated from aberrantly-instigated ISCs residing at the crypts of colon epithelium, which are responsible for propagating the entire tumor cell population and seeding distant metastases. Therefore, eliminating these TICs would rationally inhibit CRC progression and recurrence efficiently. Nonetheless, current commonly-used combinatory regimens based on fluorouracil backbone (e.g. FOLFOX and FOLFIRI) do not display ideal effect against TICs that stay quiescent most of the time and reserve strong potential of plasticity. Our study herein reported a novel strategy to eradicate TICs in CRC through KDM6 inhibition and further clarified the underlying mechanism that involves enhancer reprogramming. We also showed that KDM6B level could be a potential predictor for CRC overall prognosis and recurrence. These findings will potentially be beneficial in developing new therapeutics against CRC, optimizing current chemotherapeutic strategies for relieving resistance, and guiding better post-treatment surveillance.

In accordance with our findings, being a core member of a COMPASS (Complex Proteins Associated with Set1) -like transcription activator complex containing H3 lysine 4 methyltransferases MLL2/3 and histone acetyl transferase p300 29, KDM6A has been implicated in shaping enhancers and remodeling chromatin in developmental and physiological processes such as cell lineage determination 30 and mounting of innate immune response 31 and also in disease conditions including cancer 32, 33. Our current study identified KDM6A as a potential regulator of global enhancers, especially super-enhancers, probably working with p300 and BRD4, two key coactivators that have been shown to demarcate super-enhancer sites together and control local transcriptional outputs. However, how KDM6A orchestrate p300 and BRD4 recruitment at super-enhancers through shaping chromatin environment and whether its activity requires pre-priming of chromatin accessibility by other pioneer transcription factors need to be further explored. In addition to KDM6A, KDM6B has also been implicated in enhancer regulation in different biological processes 34, 35, but whether they act redundantly or non-redundantly in shaping enhancer states remain elusive. Interestingly, current study showed that although KDM6A and KDM6B regulate some target genes in similar manner in HCT116 cells (e.g. *ID1* and *TERT*), certain target genes such as *ID1* were responsive to *KDM6A* but not *KDM6B* silencing in another cell line HT29 (Figure 5G and Figure S2D), indicating that the mechanism-of-action of KDM6 demethylases and their functional interplay might be cell-type specific.

It has long been recognized that histone methylation is a vital player in development and disease 36. Methylation at different histone residue sites gives distinct impact on transcriptional activity and high-order chromatin structure. Proper control of H3K27me3 level and its distribution pattern across different genomic features are essential for embryonic development and maintenance of physiological homeostasis, which frequently goes awry during tumorigenesis 12, 37, 38. Therefore, the feasibility of targeting molecules keeping the balance of H3K27me3 for cancer therapeutics has been under evaluation for a long time, such as EZH2 inhibitors 14, 39. Recently, the other side of the strategy, counteracting KDM6 activities, emerged and the results are encouraging in the pre-clinical studies in some cancers. For instance, GSK-J4 strongly inhibits the growth of pediatric brainstem glioma bearing histone H3.3 K27M mutation through restoring global H3K27me3 level 15, sensitizes diffuse intrinsic pontine glioma to radiation therapy by repressing the expression of DNA repair genes 16, and induces differentiation of neuroblastoma accompanied by upregulation of apoptosis-promoting gene PUMA 18. Our study herein uncovered a novel role of GSK-J4 in eradicating TICs of CRC, which probably depends on enhancer reprogramming by GSK-J4 at key stemness-controlling genes. Interestingly, we found that super-enhancers were more sensitive to GSK-J4 treatment than typical-enhancers. Thus, it is rational to test the potential synergistic effect of combining GSK-J4 with other molecules targeting super-enhancers such as BET or CDK7 inhibitors in future.

Notably, based on intensive studies across a broad spectrum of tumors 40, 41, the functional roles of both KDM6A and KDM6B in tumorigenesis were found to be highly cell type-specific and pathologic context-dependent. For instance, KDM6B could promote TGF-β-induced epithelial-mesenchymal transition (EMT) through activating SNAI1 in breast cancer cells 42, and instigate proliferative, metastatic, and self-renewal capacities of tumor cells in liver, ovarian, and skin cancers by modulating multiple signalings 24, 43, 44. On the other side, the tumor-suppressive role of KDM6B was supported by the evidence showing that it is a key activator of the *INK4a*/*ARF* tumor suppressor locus in response to oncogenic stress such as *RAS* activation 45 and it is involved in the epigenetic regulation of cis-regulatory elements activated during DNA damage response in a p53-dependent manner 35. Therefore, the multi-faced roles of KDM6 genes highlight the importance of understanding their exact mechanism-of-action during different tumorigenic processes, which might be critical for optimizing therapeutic strategy more precisely.

## Materials and Methods

### Sphere formation assay

Dispersed single cells were cultured in serum-free Dulbecco's modified Eagle's medium DMEM/F-12 (Hyclone) supplemented with 1X B27 (ThermoFisher), 5 μg/mL insulin (ThermoFisher), 20 ng/ml fibroblast growth factor (FGF) (GenScript), and 20 ng/ml epidermal growth factor (EGF) (GenScript) in ultra-low attachment cell culture plates (Corning) for five to seven days. GSK-J4 or vehicle DMSO (dimethyl sulfoxide) at indicated concentration were added to the medium and refreshed every two to three days. The tumor spheres were photographed under a light microscope, and the numbers of spheres were counted in at least five independent fields for each well. For culturing of ALDH positive cells, cells were first stained using ALDEFLUOR kit as described below and sorted on a flow cytometry platform (BD FACSAria II). ALDH positive cells were collected and subject to sphere formation culture with or without GSK-J4 treatment.

### Flow cytometry analysis

The ALDH activity of tumor cells was determined using ALDEFLUOR kit (STEMCELL Technologies) according to manufacturer's protocol. Briefly, two million cells treated with or without GSK-J4 at indicated doses for 48 h were incubated with 5 µL of the activated ALDEFLUOR reagent for 30 min at 37 °C and were immediately subjected to analysis by a flow cytometer at FL1 channel (BD Accuri C6). For each batch of experiments, a reaction containing both activated ALDEFLUOR reagent and diethylaminobenzaldehyde (DEAB), a specific inhibitor of ALDH, was set to control for background fluorescence. For analysis of CD24^+^CD44^+^ cells, HCT116 or HT29 cells treated with or without GSK-J4 at indicated doses for 48 h were incubated with anti-CD24-FITC (ab30350, 1:20) and anti-CD44-PE/Cy7 (ab46793, 1:400) antibodies at room temperature for 30 min and analyzed immediately on a BD Accuri C6 flow cytometer.

### Mouse intestine crypt isolation and organoid culture

Mouse handling and experimental procedures in this study were approved by the Center for Laboratory Animals, School of Medicine, Shenzhen University. The small intestine of C57BL/6J wild-type mice was isolated and flushed with cold PBS (without calcium or magnesium) through a 20 ml syringe with a 19-G blunt needle. Duodenum and jejunum was opened longitudinally and cut into small fragments about 1-2 cm in length. The tissues were transferred to a 50 ml tube and washed in cold PBS with vigorous shaking for 2 min. The supernant was centrifuged for collecting the first fraction of intestine epithelium sample that mostly contains upper villi, while the rest tissues were incubated with 1 mM ethylenediaminetetraacetic acid (EDTA) in PBS for 30 min at 4 °C on a tube roller, from which the second fraction of sample containing a mixture of crypts and villi was collected. Lastly, the third fraction of sample mostly containing crypts were obtained by incubating the remaining intestine tissues with 5 mM EDTA in PBS for 1 h at 4 °C on a tube roller and centrifugation of the resulting supernant.

For organoid culture, crypts from the third fraction of intestine epithelium were first examined under a light microscope to ensure its purity and then seeded in growth factor-reduced basement matrix (BD Bioscience, 356231) in a pre-warmed culture plate. Incubate the plate in a tissue culture incubator for 15 min so the basement matrix polymerizes, followed by overlaying the matrix with sufficient volume of organoid culture medium prepared according to the following formula: advanced DMEM/F-12 medium containing 1X Glutamax, 2.5% fetal bovine serum, 10 mM Hepes, 1X penicillin/streptomycin, 1X B27 supplement, 1X N2 supplement, 1.25 mM n-Acetylcysteine, 50 ng/ml mouse EGF, 50% WRN conditioned medium produced by L-WRN cells which contains mouse Wnt3a, R-Spondin and Noggin. Liquid medium with or without GSK-J4 were refreshed every two to three days. Organoids were allowed to grow for one to two weeks until typical round cystic shape and budding structures become visible, and then photographed under a light microscope.

### Tumor xenograft model and *in vivo* imaging

HCT116 cells stably expressing firefly luciferase gene (HCT116-luc) were constructed by infected parental cells with lentivirus containing pLenti-CMV-V5-LUC-Blast plasmid (Addgene, 21474) and selected with 1 μg/ml blasticidin for two weeks. Four to six week-old female BALB/c nude mice were subcutaneously inoculated with 5×10^6^ HCT116-luc cells at their flank. When the average volume of tumors (length×width^2^ ×0.52) reached 100 mm^3^, mice were randomly divided into two groups (6 mice per group), which were subject to vehicle (12.5% DMSO + 87.5% PBS) or GSK-J4 (25 mg/kg) treatment via intraperitoneal injection every two days for 5 cycles. Ten days after starting GSK-J4 treatment, tumor-bearing mice were given a single dose of luciferin (GoldBio) at 150 mg/kg intraperitoneally and subsequently analyzed on an *in vivo* imaging system (IVIS Spectrum, PerkinElmer).

### Chromatin immunoprecipitation-sequencing (ChIP-seq) with spike-in normalization

ChIP-seq experiments were essentially carried out as described before 46 with modification in spike-in normalization. Briefly, a constant amount (about 5% of total chromatin) of chromatin from a foreign species (mouse intestine in our case) to each ChIP reaction before the immunoprecipitation step to normalize potential bias introduced by experiment and/or data processing procedures between different reactions, which could improve the power and accuracy of quantitative comparison of ChIP-seq signals 47. Please refer to Supplementary materials and methods for detailed procedures of ChIP. Antibodies used for ChIP were anti-H3K27ac (Abcam, ab177178), anti-H3K27me3 (Cell Signaling Technology, 9733), and anti-KDM6A (Bethyl Lab, A302-374A). All antibodies were used in a dilution of 1:200 in ChIP reactions.

For library construction, ChIP's DNA was subjected to end-repair and then was 3' adenylated. Adaptors were ligated to the ends of these 3' adenylated fragments. Fragments were amplified by PCR and PCR products were purified and selected with the Agencourt AMPure XP-Medium kit. The double stranded PCR products were heat denatured and circularized by the splint oligo sequence. The single strand circle DNA (ssCir DNA) were formatted as the final library. Final library was qualified by Qubit ssDNA kit and sequenced on a BGISEQ-500 platform (Beijing Genomics Institute).

### ChIP-seq data analysis

Raw reads were aligned to reference genome hg19 by Bowtie2 (v. 2.2.9). Around 20 million clean reads were obtained for H3K27ac and KDM6A samples, while around 50 million clean reads were generated for H3K27me3 samples due to its high noise background. For spike-in normalization, the mapped (primary aligned) reads of the BAM file was processed by samtools for two reference genomes hg19 and mm10 respectively, then the bamCoverage in deeptools (v 3.3.0) was used to generate the bigwig files with the normalization scale factor. Peak calling from alignment results were performed by MACS2 (v. 2.1.6) with default parameters expect p<1e-09, no lambda, no model, and broad peak were used for H3K27me3 samples. Blacklist regions from ENCODE project were filtered out. The bedGraph files generated from MACS2 were converted to bigwig files with ucsc bedGraphToBigWig tool. Integrative Genomics Viewer (IGV) was used to visualize peaks across the genome.

For super-enhancer analysis and gene-to-enhancer annotation, the ROSE (Rank Ordering of Super-Enhancers) algorism 48 with parameters -s 12500 -t 2000, in which enhancers within 12.5 kb are stitched together, were used. Enhancers above the inflection point of the ranking curve were defined as super-enhancers. For identification of H3K27me3 domains which typically correspond to large genomic spans covered with continuous H3K27me3 signal, H3K27me3 peaks within 2 kb to each other were merged and merged peaks larger than 5 kb were defined as H3K27me3 domains 49. For ChIP signal heatmap and profile curve in given genomic intervals, score matrix was first calculated by computeMatrix in deeptools (v. 3.1.3) at a bin size of 10 bp, then plotHeatmap and plotProfiles in deeptools were used for visualization.

### Quantitative reverse transcription-PCR (qRT-PCR)

HCT116 or HT29 cells at a confluency of around 80% were treated with vehicle (DMSO) or GSK-J4 at indicated concentration for 48 h before harvest. Total RNA was extracted by TRIzol reagent (Life Technologies) according to manufacturer's instructions. Reverse transcription was performed using PrimeScript RT kit with genomic DNA eraser (Takara), and qPCR was conducted using SYBR Green PCR Master Mix (Takara) on a qTOWER platform (Jena). Human or mouse *GAPDH* gene was used as an internal control. Primers used in this study were listed in Supplementary Table S1.

### RNA sequencing (RNA-seq)

Total RNA was extracted and mRNA was enriched for library construction. Qualified library was sequenced on MGISEQ-2000 platform (Beijing Genomics Institute). Raw reads were cleaned using SOAPnuke (v.1.4.1), generating around 22 million clean reads per sample. Hisat (v. 0.1.6) and Bowtie2 (v. 2.3.5) were used to align the clean data to reference genome (hg19) and gene respectively. Transcript abundance was quantified by HTseq (v. 0.11.2) and differentially expressed genes (DEGs) were identified by the edgeR package from the R software. Genes with fold change ≥ 2 and false discovery rate (FDR) ≤ 0.01 were considered as significant DEGs.

### Gene Set Enrichment Analysis (GSEA)

GSEA of RNA-seq data was performed essentially as originally described using the pre-ranked method 50. All genes differentially expressed between two samples were ranked according to the absolute value of Log_10_ (FDR) with the sign of fold change. The pre-ranked gene list was then loaded for analysis. Number of permutations was set to 1000 as default. Gene sets analysed in this study were from Molecular Signatures Database (MSigDB), except the following gene sets, CRC proliferation signature 5, ISC and LGR5-refined ISC signatures 5, and differentiated enterocytes signature 51, which were reported elsewhere.

### Western blotting

Western blotting was performed as described previously 52. Briefly, 50 microgram of total protein was loaded for each sample. Primary antibody incubation was performed at 4 °C overnight with gentle shaking, and secondary antibody was incubated with membrane at room temperature for 1 h. Antibodies used in this study were anti-KDM6A (Bethyl Lab, A302-374A, 1:4000), anti-KDM6B (Abcam, ab38113, 1:1000), anti-β-Catenin (Abcam, ab32572, 1:2000), anti-MYC (Cell Signaling Technology, 18583, 1:1000), anti-AXIN2 (Proteintech, 20540-1-AP, 1:2000), and anti-β-actin (Sigma, A2228, 1:5000).

### Kaplan-Meier survival analysis and ROC curve plotting

Expression datasets of CRC samples with clinical outcome information from several independent studies were obtained from TCGA data portal 53 or GEO database (GSE39582, GSE41258, GSE17538) 54-56. Optimal cutpoint for *KDM6A* or *KDM6B* expression level used for grouping patient samples in each dataset was calculated by the maxstat package in R. For microarray datasets with multiple probes for the same gene, mean expression of all probes were used. Kaplan-Meier Survival curves were plotted using the survminer package in R for both overall survival and relapse-free survival. Log-Rank test was used to evaluate statistical significance and *P* < 0.05 was considered to be significant. For generating receiver operating characteristic (ROC) curve, the plotROC package in R was used.

### Data availability

ChIP-seq and RNA-seq data generated in this study have been deposited at the Gene Expression Omnibus (GEO) under accession number GSE146679.

For more information about materials and methods, please refer to Supplementary materials and methods.

## Supplementary Material

Supplementary methods, figures and table.

## Abbreviations

CRC: colorectal cancer
TICs: tumor-initiating cells
ISCs: intestinal stem cells
PRC2: polycomb complex 2
ESCs: embryonic stem cells
EDTA: ethylenediaminetetraacetic acid
ChIP-seq: chromatin immunoprecipitation-sequencing
qRT-PCR: quantitative reverse transcription polymerase chain reaction
shRNA: short hairpin RNA
GSEA: Gene Set Enrichment Analysis
NES: normalized enrichment score
ChIA-PET: chromatin interaction analysis with paired-end tag sequencing
ROC: receiver operating characteristic
CI: combinatory index
FDR: false discovery rate
n.s.: non-significant
SE: super-enhancer
TE: typical-enhancer
PCC: Pearson correlation coefficient
