# Supplementary material for: Targeted inhibition of KDM6 histone demethylases eradicates tumor-initiating cells via enhancer reprogramming in colorectal cancer
Source: Theranostics. 2020 Aug 8;10(22):10016–30. doi: 10.7150/thno.47081 (PMC7481431; doi:10.7150/thno.47081)
Supplement: Supplementary file 1 — Supplementary methods, figures and table. [file thnov10p10016s1.pdf]

Supplementary materials and methods

Reagents, cell lines and shRNA plasmids

GSK-J4 powder were purchased from MedChemExpress (HY-15648B) and dissolved in DMSO (dimethyl sulfoxide) for in intro cell line experiments and dissolved in 12.5% DMSO plus 87.5% PBS for in vivo mouse experiments. For reagents used for cell and organoid culture, RPMI1640 medium and DMEM medium (high glucose) were from Hyclone; advanced DMEM/F-12, fetal bovine serum, Glutamax, penicillin/streptomycin, insulin, B27 supplement, N2 supplement were from ThermoFisher; human and mouse EGF and FGF were from GenScript.

Four human CRC cell lines (HCT116, HT29, SW480, RKO) and one murine CRC cell line CT26 were obtained from cell bank of Chinese Academy of Sciences and maintained in RPMI1640 medium supplemented with 10% fetal bovine serum in a 5% CO_2 incubator at 37 ℃. L-WRN cells were obtained from ATCC (American Type Culture Collection) and maintained in DMEM medium (high glucose) supplemented with 10% fetal bovine serum. LentiX-293T cell line used for virus production were purchased from Clontech and maintained in DMEM medium (high glucose) supplemented with 10% fetal bovine serum. Cell lines used in this study were authenticated by STR profiling and tested routinely for mycoplasma contamination.

For RNAi experiments, plasmids containing shRNAs targeting KDM6A (5’ GCAGATACATGGTGTTCAA 3’) or KDM6B (5’ GCCGAATTCAAGATCCTAC 3’) were constructed based on a pLVX-ZsGreen-Puro vector backbone. A non-targeting construct were used as control. All constructs were confirmed by Sanger sequencing.

Cell proliferation assay

Dispersed cells were seeded in 96-well plate (2,000 to 5,000 cells per well). 24 h after

seeding, GSK-J4 or vehicle (DMSO) of indicated concentration(s) were added, and CCK8 (Cell Counting Kit-8, Dojindo) reagent was added to cell culture at indicated time points and absorbance at 450 nm was measured after one hour of incubation.

Colony formation assay

For colony formation assay using monolayer culture, cells were seeded in 6-well plate (1000 cells per well). Two days after cell seeding, GSK-J4 or vehicle (DMSO) of indicated concentration(s) were added and the medium were refreshed every two to three days. Cells were allowed to grow for around two weeks and the colonies were stained by crystal violet. The number of colonies was counted by IMAGE J software. Colony formation capacity was calculated by formula: colonies number / initial cell number.

Transwell migration assay

Cells in serum-free medium were seed in the upper chamber of transwell insert (Corning) in 24-well plate (2×10^5 cells per well), with medium containing 10% fetal bovine serum in the bottom chamber. Medium containing GSK-J4 or DMSO were changed 6 hours after seeding. After thirty hours of incubation, migrated cells at the bottom side of the insert membrane were stained with crystal violet. At least five random fields were photographed and the number of migrated cells was counted by IMAGE J software.

Wound healing assay

Cells were seeded at a density of 95% in six-well plate (Corning). 12 h later, the wound was created with a 20 μl pipette tip with a straight scratch. Cells were continuously cultured in serum-free medium with or without GSK-J4 for 36 h and photographed under a microscope at indicated time points. The distance between the wound was measured by IMAGE J software. The cell mobility potential was calculated using the following

formula: (wound distance at initial time - wound distance at current time) / wound distance at initial time.

Chromatin immunoprecipitation (ChIP)

HCT116 cells at a confluency of around 80% were treated with vehicle or 15 µm GSK-J4 for 48 h before fixation for ChIP. After treatment, Cells cross-linked in 1% formaldehyde for 10 min (1 × 10^7 cells per reaction) were harvested and washed once with Farnham Lysis buffer (5 mM PIPES, 85 mM KCl, 0.5% NP-40, pH 8.0). The resulting nuclei were resuspended in ChIP RIPA buffer (1% NP-40, 0.5% sodium deoxycholate, 0.1% SDS in PBS, pH 7.4). Chromatin was fragmented by sonication to 200 to 500 bp in size and incubated with primary antibody in or control IgG pre-conjugated with Protein G magnetic beads (Pierce) at 4 °C overnight. 1% of sheared chromatin was saved as input. The beads were washed five times with LiCl wash buffer (0.1 M Tris, 0.5 M LiCl, 1% NP-40, 1% sodium deoxycholate, pH 7.5) followed by a single wash with TE buffer (10 mM Tris-HCl, 0.1 mM Na_2EDTA, pH 7.5). Chromatin was eluted by incubating the beads with IP elution buffer (1% SDS, 0.1 M NaHCO3) at 65 °C for 1 h. Cross-links were reversed by incubating eluted and input chromatin at 65 °C for 16 h. Subsequently, DNA was purified with QIAquick PCR Purification Kit (Qiagen).

Lentiviral particle production and stable cell line generation

Lentiviral particles were produced using a 3rd generation packaging system in HEK293T cells. Medium was collected 72 h after transfection and viruses were concentrated using Lenti-X Concentrator reagent (Clontech). Cells were seeded in 6-well pate one day before infection, and were infected by the lentiviral particles in the presence of 4 µg/ml polybrene. 2 µg/ml puromycin was applied 72 h post-infection for stable cell line selection.

Statistical analysis

Data were presented as mean ± standard error of the mean (SEM). Unless specified elsewhere, all experiments in this study were repeated in triplicate independently. Difference between two independent groups was analysed by unpaired *t*-test under two-tail hypothesis. Difference of multiple groups was evaluated by one-way analysis of variance (ANOVA) and Bonferroni post hoc tests were used to further evaluate results with significance from ANOVA. Kaplan–Meier survival analysis was used to test the association of gene expression level with patient prognostic outcome. Statistical analysis were performed in GraphPad Prism 7 or using SPSS 21.0 package.

Supplementary figures

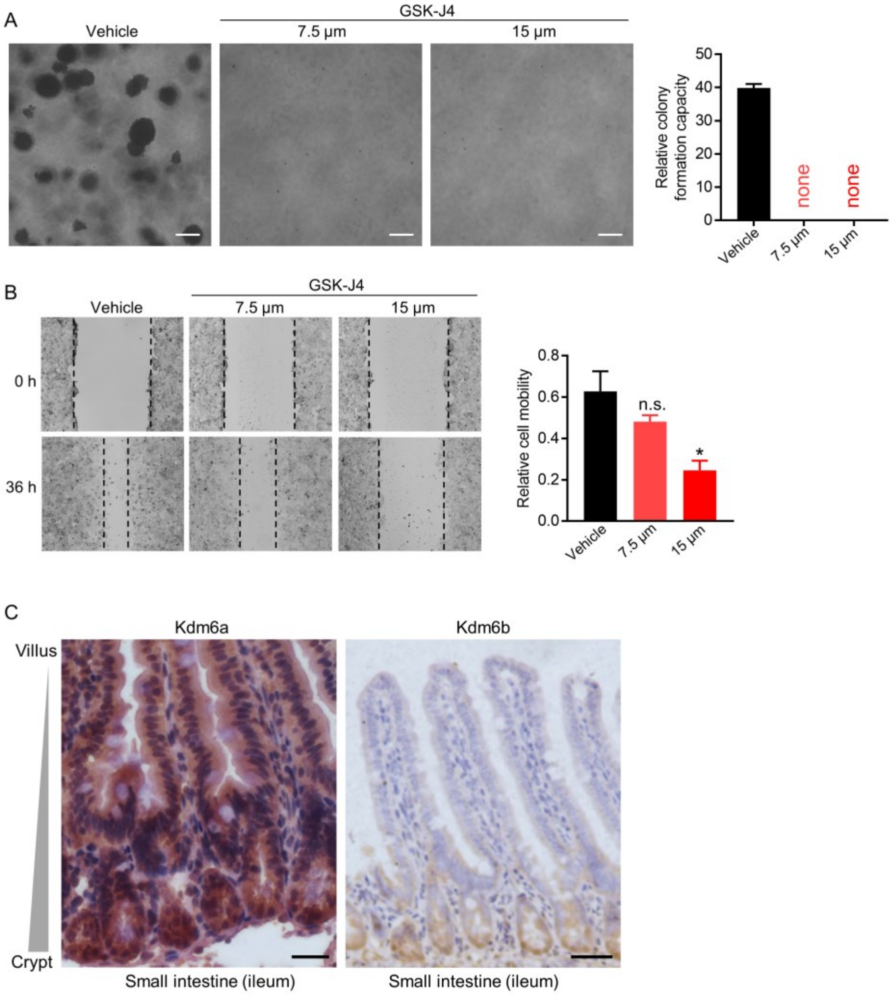

Supplementary Figure 1. Soft-agar colony formation assay (A) and wound healing assay (B) in HCT116 cells treated with or without GSK-J4 at indicated doses. Scale bar represents 100 μm. * $p < 0.05$; n.s., non-significant. (C) Immunohisochemistry analysis of Kdm6a (left) and Kdm6b (right) expression in the small intestine (ileum part) from wild-type C57-BL/6 mice. Scale bar represents 100 μm.

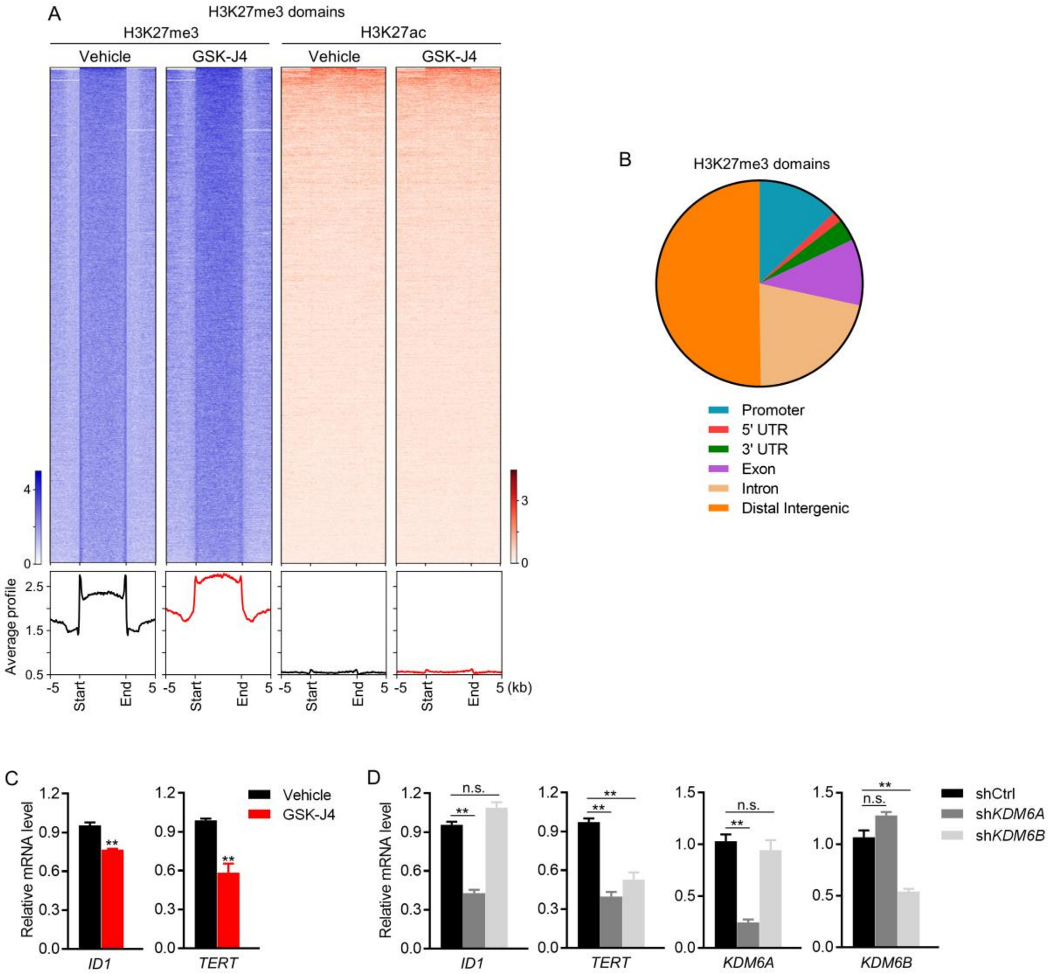

Supplementary Figure 2. (A) Heatmaps and average profile curves of H3K27me3 and H3K27ac ChIP-seq data across a 10 kb window centered at standardized H3K27me3 domains. (B) Pie chart showing the distribution of H3K27me3 domains across different genomic features. UTR, untranslated region. (C) mRNA level of *ID1* and *TERT* were evaluated by qRT-PCR in HT29 cells treated with or without 20 µm GSK-J4 for 48 hours. (D) Expression level of indicated genes was examined by qRT-PCR in HT29 cells 72 hours after infection with lentivirus containing shRNA targeting *KDM6A* or *KDM6B*. A non-targeting sequence cloned into the same backbone (shCtrl) was used as control. ** $p < 0.01$; n.s., non-significant.

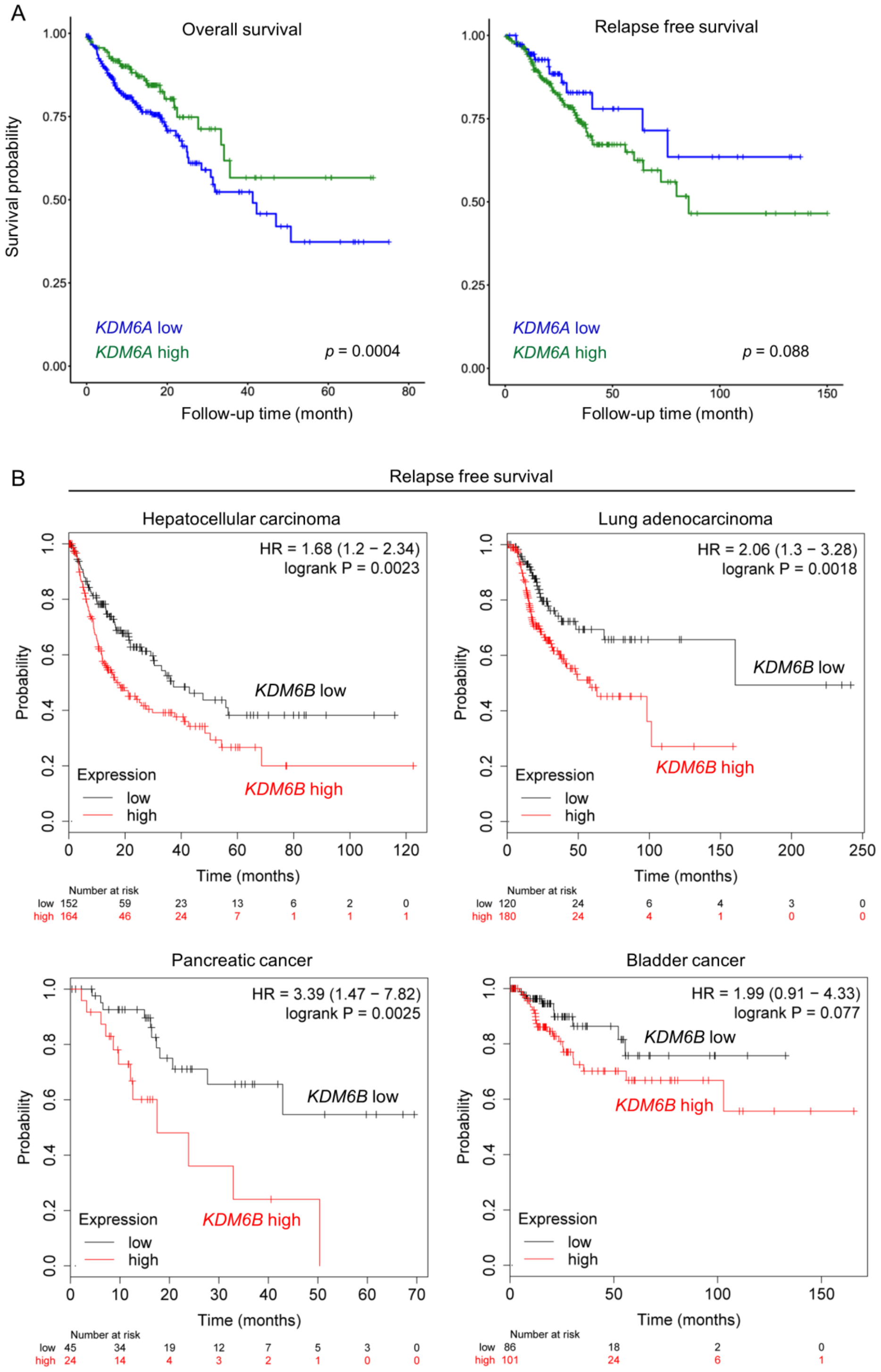

Supplementary Figure 3. (A) Kaplan–Meier survival curves showing association of *KDM6A* level with overall survival and relapse-free survival of CRC patients in TCGA cohort. Significance was determined by Log-Rank test. (B) Association of *KDM6B* level with relapse-free survival of four different cancer types was examined by Kaplan–Meier survival analysis using KM-plotter (http://kmplot.com/). HR, hazard ratio.

Supplementary Table 1. Primers used in this study

Primer name	Organism	Sequence (5' to 3')	Amplicon size (bp)
KDM6A-F	Human	TTCCTCGGAAGGTGCTATTCA	241
KDM6A-R	Human	GAGGCTGGTTGCAGGATTCA	
KDM6B-F	Human	CGCTGCCTCACCCATATCC	187
KDM6B-R	Human	ATCCGCGACCTCTGAACTCT	
GAPDH-F	Human	GGAGCGAGATCCCTCCAAAAT	197
GAPDH-R	Human	GGCTGTTGTCATACTTCTCATGG	
SOX4-F	Human	AGCGACAAGATCCCTTTCATTC	109
SOX4-R	Human	CGTTGCCGGACTTCACCTT	
TERT-F	Human	AAATGCGGCCCCTGTTTCT	76
TERT-R	Human	CAGTGCGTCTTGAGGAGCA	
AXIN2-F	Human	CAACACCAGGCGGAACGAA	103
AXIN2-R	Human	GCCCAATAAGGAGTGTAAGGACT	
MYC-F	Human	GGCTCCTGGCAAAAGGTCA	
MYC-R	Human	CTGCGTAGTTGTGCTGATGT	119
ID1-F	Human	CTGCTCTACGACATGAACGG	124
ID1-R	Human	GAAGGTCCCTGATGTAGTCGAT	
NANOG-F	Human	TTTGTGGGCCTGAAGAAAACT	116
NANOG-R	Human	AGGGCTGTCCTGAATAAGCAG	
EPCAM-F	Human	AATCGTCAATGCCAGTGTACTT	178
EPCAM-R	Human	TCTCATCGCAGTCAGGATCATAA	
Kdm6a-F	Mouse	CGGGCGGACAAAAGAAGAAC	203
Kdm6a-R	Mouse	CATAGACTTGCATCAGATCCTCC	
Kdm6b-F	Mouse	TGAAGAACGTCAAGTCCATTGTG	142
Kdm6b-R	Mouse	TCCCGCTGTACCTGACAGT	
Gapdh-F	Mouse	AGGTCGGTGTGAACGGATTTG	95
Gapdh-R	Mouse	GGGGTCGTTGATGGCAACA	
Lgr5-F	Mouse	GGACCAGATGCGATACCGC	194
Lgr5-R	Mouse	CAGAGGCGATGTAGGAGACTG	
Olfm4-F	Mouse	CAGCCACTTTCCAATTTCACTG	175
Olfm4-R	Mouse	GCTGGACATACTCCTTCACCTTA	
Axin2-F	Mouse	ATGAGTAGCGCCGTGTTAGTG	150
Axin2-R	Mouse	GGGCATAGGTTTGGTGGACT	
Krt20-F	Mouse	GTGGCTCGCTGTATAGGAAGG	146
Krt20-R	Mouse	CAGGTCCGATCCGTTGGAG	
Fabp2-F	Mouse	GTGGAAAGTAGACCGGAACGA	117
Fabp2-R	Mouse	CCATCCTGTGTGATTGTCAGTT	
